# Supplementary material for: Intracycle Velocity Variation in Swimming: A Systematic Scoping Review
Source: Bioengineering (Basel). 2023 Feb 28;10(3):308. doi: 10.3390/bioengineering10030308 (PMC10044880; doi:10.3390/bioengineering10030308)
Supplement: Supplementary file 1 [file bioengineering-10-00308-s001.zip › Evidence Gap Map.html]

EPPI-Mapper


X

- Filters
- Hide Headers
  Show Headers
- Fullscreen
  Exit Fullscreen
- About
- Submit a Study
- View Records

Generated using v.2.2.3 of the EPPI-Mapper
powered by EPPI Reviewer
and created with


by the
Digital Solution Foundry team.
